# Cost-utility analysis of deep learning-based opportunistic osteoporosis screening using chest radiographs: implications of diagnostic thresholds and confirmatory testing

**DOI:** 10.1007/s11657-026-01788-5

**Published:** 2026-10-23

**Authors:** Bo-Xiang Shi, Dung-Jang Tsai, Chin Lin, Shih-Hua Lin, Ping-Hsuan Hsieh, Wen-Hui Fang

**Affiliations:** 1 School of Pharmacy, College of Pharmacy, National Defense Medical University, Taipei, Taiwan, ROC; 2 Department of Pharmacy Practice, Tri-Service General Hospital, National Defense Medical University, Taipei, Taiwan, ROC; 3 Digital Medical Center, Tri-Service General Hospital, National Defense Medical University, Taipei, Taiwan, ROC; 4 School of Medicine, College of Medicine, National Defense Medical University, Taipei, Taiwan, ROC; 5 Division of Nephrology, Department of Internal Medicine, Tri-Service General Hospital, National Defense Medical University, Taipei, Taiwan, ROC; 6 Department of Nephrology, School of Medicine, College of Medicine, National Defense Medical University, Taipei, Taiwan, ROC; 7 Department of Family and Community Medicine, Tri-Service General Hospital, National Defense Medical University, Taipei, Taiwan, ROC

**Keywords:** Osteoporosis, Deep learning, Cost-effective, Chest radiography, Opportunistic screening

## Abstract

***Summary*:**

Osteoporosis often goes undetected until a fracture occurs. Using a deep learning model to analyze routine chest radiographs may cost-effectively identify at-risk individuals. Economic benefit depends on how common the disease is, how the model is calibrated, and whether patients follow through with confirmatory bone density testing and pharmacological treatment initiation.

**Background:**

Osteoporosis is common among postmenopausal women and often unrecognized until fragility fractures occur, despite its clinical and economic burden. Deep learning (DL)-based opportunistic screening using existing chest radiographs is a potential approach, but its economic value may vary with disease prevalence, diagnostic performance, and care pathway completion.

**Aim:**

To assess the economic value of a DL-based opportunistic osteoporosis screening approach among Taiwanese postmenopausal women.

**Methods:**

A cost-utility analysis was conducted from the Taiwan healthcare perspective using a hybrid decision tree and Markov model. The model simulated a cohort of 50-year-old postmenopausal women over a 50-year horizon. Model inputs, including diagnostic performance, long-term outcomes, costs, and utilities, were derived from published literature, preferably local sources. Additional analyses examined alternative prevalence settings, sensitivity and specificity combinations, and confirmatory DXA and treatment initiation rates.

**Results:**

In the base case, assuming full care-pathway completion, DL-based screening was cost-effective versus no screening, with an ICER of 21,827 per QALY, below the willingness-to-pay threshold of one GDP per capita in Taiwan. The approach became cost-effective when osteoporosis prevalence exceeded 14.3%. Across the diagnostic continuum, a sensitivity of 86.6% and specificity of 76.3% generated the greatest economic benefit.

**Conclusion:**

DL-based opportunistic osteoporosis screening was cost-effective under full care-pathway implementation. Its value depended on disease prevalence, diagnostic performance, and completion of confirmatory DXA and treatment initiation, and was not maintained under real-world examination estimates observed in Taiwan. Strengthening the post-screening care pathway is therefore central to realizing this value.

## Introduction

Osteoporosis is a systemic skeletal disorder characterized by reduced bone mineral density, leading to an increased risk of fragility fractures [1]. This condition predominantly affects postmenopausal women and often remains undiagnosed until fractures occur, owing to its asymptomatic progression. These fractures substantially reduce quality of life and generate a significant economic burden [2]. In 2018, the global cost of osteoporosis treatment was estimated at approximately $5.3 million, accounting for nearly 20% of gross domestic product (GDP) [3].

Pharmacological intervention with anti-osteoporotic medication plays a pivotal role in fracture prevention by increasing bone mineral density and reducing the risk of subsequent fractures [4]. While bisphosphonates and denosumab are the most commonly prescribed agents in Taiwan, their reimbursement under the National Health Insurance (NHI) is strictly regulated to patients meeting specific severity criteria, such as a T-score ≤ -2.5 combined with a history of fragility fractures [5].

Early detection and timely diagnosis are paramount to initiating appropriate preventive strategies before a fracture occurs. Currently, the measurement of bone mineral density using dual-energy X-ray absorptiometry (DXA) serves as the gold standard for diagnosing osteoporosis [5]. However, despite its diagnostic accuracy, DXA examination remains underutilized due to limited accessibility, cost, and the lack of universal screening mandates [6]. This diagnostic gap highlights an unmet need for alternative and more accessible screening approaches.

Several validated models have demonstrated consistently high diagnostic accuracy across independent populations, supporting the growing international maturity of this technology [7–11]. For instance, Tsai’s model was developed and validated using a large local dataset in Taiwan and was subsequently evaluated in a randomized clinical trial conducted in real-world clinical settings, in which the DL screening group exhibited higher rates of osteoporosis detection [12, 13].

DL-based opportunistic osteoporosis screening has shown favorable cost-effectiveness results in multiple countries and healthcare settings [14–18]. However, the economic value of screening is highly context-dependent, varying with fracture risk profiles, osteoporosis prevalence, downstream treatment patterns, and healthcare system financing [19]. Moreover, cost-effectiveness results depend on the DL model’s diagnostic performance, which determines the balance between true positives and false positives and, consequently, the intensity of confirmatory testing and treatment [20–22]. Therefore, in this study, we aimed to evaluate the economic value of the DL screening approach for early detection of osteoporosis among Taiwanese postmenopausal women. We further evaluated how variations in osteoporosis prevalence and the diagnostic performance of the DL model influenced the economic value of DL-based opportunistic screening.

## Methods

### Model conceptualization

A hybrid decision tree and Markov model was developed to evaluate the economic value of opportunistic osteoporosis screening among postmenopausal women in Taiwan. The DL model adopted in this study was developed and validated on chest radiographs obtained during routine clinical care at a medical center in Taiwan, and was prospectively evaluated in the OPSCAN randomized controlled trial, in which the screening workflow was applied to patients receiving chest radiography for indications other than osteoporosis [12, 13]. The modeled screening pathway was aligned with the clinical scenario of the OPSCAN trial [13]. Specifically, the DL model was assumed to be applied to existing CXRs that had already been obtained during routine clinical care, rather than to newly acquired CXRs ordered for osteoporosis screening. The analysis was performed from the Taiwan healthcare perspective.

**Fig. 1:**
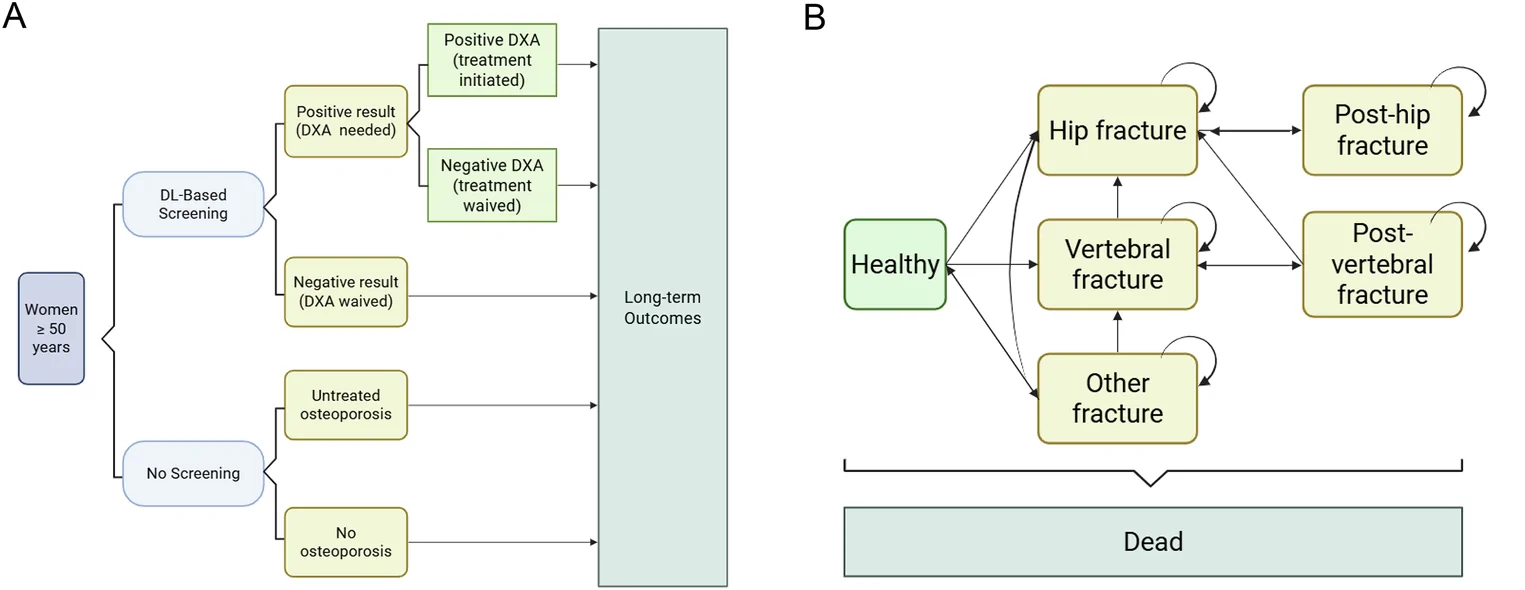
**A** Decision tree of evaluating the economic value of opportunistic osteoporosis screening using deep learning-based (DL-based) chest radiographs in Taiwanese postmenopausal women. **B** Markov model for long-term outcomes of osteoporotic fracture in Taiwanese postmenopausal women. Abbreviations: CXR, chest radiograph; DL, deep learning; DXA, dual-energy X-ray absorptiometry

The decision tree partitioned the cohort into two distinct strategies: DL-based screening and the no-screening comparator (Fig. 1A). In the no-screening arm, no initial diagnostic or therapeutic intervention was applied. In contrast, individuals assigned to the intervention arm underwent initial risk stratification using the DL-based screening approach. Individuals with positive DL screening results were assumed to be notified or referred for confirmatory DXA, and pharmacological treatment was initiated only among those with DXA-confirmed osteoporosis. Because the intervention repurposed existing CXRs, the model did not include additional radiation exposure or a separate cost for ordering a new CXR solely for osteoporosis screening. Individuals with false-positive screening results underwent additional DXA examination that excluded osteoporosis and were subsequently classified as part of the general population. Conversely, individuals with confirmed osteoporosis initiated pharmacotherapy. Based on clinical expert input, 75% of treated individuals were assumed to receive denosumab, while the remaining patients received bisphosphonates. The base-case analysis assumed that all individuals with positive DL screening results completed confirmatory DXA and that all individuals with DXA-confirmed osteoporosis initiated pharmacological treatment. Lower DXA examination and treatment initiation rates were evaluated in scenario analyses.

Following initial management, all individuals entered the Markov model (Fig. 1B) to simulate long-term clinical and economic outcomes. The structure of the Markov model was validated in several published studies and comprised seven distinct health states [23–25]. The simulation modeled a cohort of 50-year-old women over a 50-year time horizon to capture lifetime outcomes. A cycle length of 1 year was adopted, consistent with the input data. In the Markov model, the occurrence of hip, vertebral, and other fragility fractures was simulated. All individuals entered the model in a well state and, during each cycle, were at risk of sustaining a fracture or death; following one year in hip or vertebral fracture states, they transitioned to corresponding post-fracture states, whereas those experiencing other fractures returned to the well state after one year.

### Model Inputs for the decision tree and Markov model

Parameters regarding fracture incidence, mortality, and direct medical costs were obtained from domestic sources [26–28]. For individuals with DXA-confirmed osteoporosis, untreated fracture incidence from Lai et al. was used to define the underlying fracture risk [26]. Adjusted hazard ratios from Fu et al. were used to represent differences in fracture hazard according to medication class and treatment persistence [29]. Drug-specific hazards were calculated separately for denosumab and oral bisphosphonates and were combined according to the assumed treatment distribution of 75% denosumab and 25% oral bisphosphonates. The resulting hazard was converted into the corresponding annual fracture probability.

The cost of per DL-based screening was assumed to be $6.45, equivalent to one CXR. The cost of DXA examination was sourced from the NHI pricing dataset in June 2025. Fracture-related costs were modeled using two separate components. The anti-osteoporosis medication cost was represented as an age-specific annual expenditure, and treatment was assumed to continue in the treated or post-fracture health states for up to 10 years. The other direct medical cost, excluding medication, represented reimbursed inpatient and outpatient expenditure incurred during the three years following fracture, including acute fracture management and subsequent care, and was estimated separately to avoid omission or double counting. Utility values were synthesized from the literature, with preference given to data from Taiwanese or Asian cohorts to ensure relevance to the Taiwan setting. All costs are reported in 2025 USD with a conversion rate of 31 TWD to 1 USD, and an annual discount rate of 3% was applied to both costs and effectiveness outcomes, in accordance with the health economic evaluation guidelines in Taiwan [30]. Key model parameters are detailed in Table 1.

**Table 1:** Key base-case model input parameters for the decision tree and Markov model

Description	Value	Uncertainty	Source
**Transition probabilities**			
Baseline annual fracture probability (fixed)	0.017 (hip) 0.017 (vertebral) 0.004 (other)	0.016-0.019 (hip) 0.015-0.018 (vertebral) 0.003-0.005 (other)	Lai [26]
**Mortality (untreated)**			
Mortality from healthy state	0.39% to 38.47% (increase with age)	Uniform	Taiwan Life Table
Mortality from hip fracture state	0.010 to 0.186 (increase with age)	0.002 to 0.013	Chang [27]
Mortality from vertebral fracture state	0.005 to 0.161 (increases with age)	0.001 to 0.014	Chang [27]
Mortality from other fracture states	0.006 to 0.146 (increases with age)	0.002 to 0.027	Chang [27]
Mortality from post-hip state	0.007 to 0.210 (increases with age)	0.001 to 0.015	Chang [27]
Mortality from post-vertebral state	0.005 to 0.155 (increases with age)	0.001 to 0.014	Chang [27]
**Costs (inflated to 2025 USD)**			
Anti-osteoporosis medication cost			
Hip fracture state	143.84 to 183.35 (age-dependent)	3.19 to 7.52	Chang [27]
Vertebral state	66.06 to 117.87 (age-dependent)	1.02 to 6.24	Chang [27]
Other fracture state	103.71 to 184.00 (age-dependent)	3.84 to 14.55	Chang [27]
Post-hip fracture state	71.39 to 117.13 (age-dependent)	3.05 to 7.80	Chang [27]
Post-vertebral fracture state	44.48 to 96.58 (age-dependent)	0.97 to 8.32	Chang [27]
Other direct medical cost excluding medication (post-hip and post-vertebral states)	1021.27 to 1082.5 (age-dependent)	416.7 to 437.7	Wang [28]
Costs of DXA	19.35	Uniform	NHI database [39]
Cost of per DL model screening	6.45	Uniform	Assumed to be the cost of CXR
**Utilities**			
Utility of healthy population			
55–59 years	0.772	Uniform	Si [40]
60–64 years	0.728	Uniform	Si [40]
65–69 years	0.702	Uniform	Si [40]
70–74 years	0.685	Uniform	Si [40]
75–79 years	0.669	Uniform	Si [40]
80–84 years	0.655	Uniform	Si [40]
85+ years	0.643	Uniform	Si [40]
Utility multiplier for hip fracture state	0.776	0.720–0.844	Si [41]
Utility multiplier for vertebral fracture state	0.724	0.667–0.779	Si [41]
Utility multiplier for other fracture state	0.99	0.960–1.000	Si [41], Expert opinion
Utility multiplier for post-hip fracture	0.855	0.800–0.909	Si [41]
Utility multiplier for post-vertebral fracture	0.868	0.827–0.922	Si [41]
**Other parameters**			
Sensitivity of DL model	92.90%	Uniform	Tsai [12]
Specificity of DL model	68.10%	Uniform	Tsai [12]
Age-standardized prevalence of osteoporosis in women	36.00%	Uniform	Chen [34]
Percentage receiving denosumab treatment	75%	Uniform	Expert opinion
Adjusted HR for hip fracture and other fractures during bisphosphonate treatment	1.27	1.08-1.48	Fu [29]
Adjusted HR for vertebral fracture during bisphosphonate treatment	0.99	0.83-1.18	Fu [29]
Adjusted HR for hip fracture and other fractures during denosumab treatment	0.91	0.89-0.94	Fu [29]
Adjusted HR for vertebral fracture during denosumab treatment	0.46	0.43-0.50	Fu [29]

*CXR* chest radiograph, *DL* deep-learning, *DXA* dual-energy X-ray absorptiometry, *HR* hazard ratio, *NHI* National Health Insurance

### Diagnostic performance and different prevalence settings

Because prevalence estimates derived from claims databases may be underestimated owing to undiagnosed cases [31], we examined alternative osteoporosis prevalence levels to identify the settings in which the screening approach would be considered cost-effective and to assess the robustness of the findings across different epidemiological conditions. The full diagnostic continuum of the DL model was evaluated to examine the balance between false positive results and missed osteoporosis cases. Sensitivity and specificity pairs were generated by varying the classification threshold along the receiver operating characteristic curve while holding overall discriminatory performance constant. After duplicate rounded pairs were removed, 371 unique diagnostic operating points were assessed. Missed cases were defined as false negatives and calculated as **cohort size × osteoporosis prevalence × (1 − sensitivity)**.

### Expected outcomes

The primary outcome measure was the incremental cost-effectiveness ratio (ICER), defined as the additional cost per quality-adjusted-life-year (QALY) gained. Because there is no official willingness-to-pay (WTP) threshold for economic evaluations in Taiwan, the base-case analysis adopted the WTP threshold of $39,492, corresponding to one gross domestic product (GDP) per capita in Taiwan in 2025, as a conservative reference threshold for interpreting the ICER. Interventions with an ICER below this threshold were considered economically favorable. To quantify the magnitude of economic value, the net monetary benefit (NMB) was also calculated using the following formula: **NMB** = **WTP × ΔQALY – Δcost** [32]. While the ICER reflects the additional cost per QALY gained, the NMB represents the net economic value of the intervention at a given WTP threshold, with higher values indicating greater economic value.

### Robustness of the analysis

The robustness of the analysis was assessed through multiple sensitivity analyses. Deterministic one-way analysis varied costs by ±20% and other parameters by ±10% to isolate the impact of individual inputs on the ICER. A probabilistic sensitivity analysis (PSA) was also performed to evaluate overall parameter uncertainty. Parameters were sampled from their respective probability distributions in 5000 Monte Carlo simulations, with results plotted on a cost-effectiveness plane.

To reflect incomplete progression through the downstream care pathway, we conducted a two-way scenario analysis varying both confirmatory DXA examination after a positive DL screening result and pharmacological treatment initiation among patients with DXA-confirmed osteoporosis. The confirmatory DXA examination rate varied from 10% to 100%, including the OPSCAN-observed rate of 14.6%, while the pharmacological treatment initiation rate varied from 20% to 100%, including the Taiwan-based estimate of 28.7% [13, 33]. The 100% values for both parameters represented the base-case assumption of full completion of confirmatory testing and treatment initiation, whereas lower values were used to reflect incomplete real-world progression through the post-screening care pathway.

## Results

### Base-case scenario

Using a sensitivity of 92.9% and a specificity of 68.1% in the base-case scenario, the analysis indicated that, in a simulated cohort of 1000 individuals, the DL-based screening approach generated 1194 additional life-years compared with the no-screening arm. When converting the result into cost and quality outcomes, the screening approach resulted in a higher average cost of $12,776 compared with $1226, as well as a higher average QALY value of 26.99 compared with 26.46. The resulting ICER was $21,827, which did not exceed the WTP threshold of one GDP per capita and was therefore considered cost-effective.

### Diagnostic performance and different prevalence settings

**Fig. 2:**
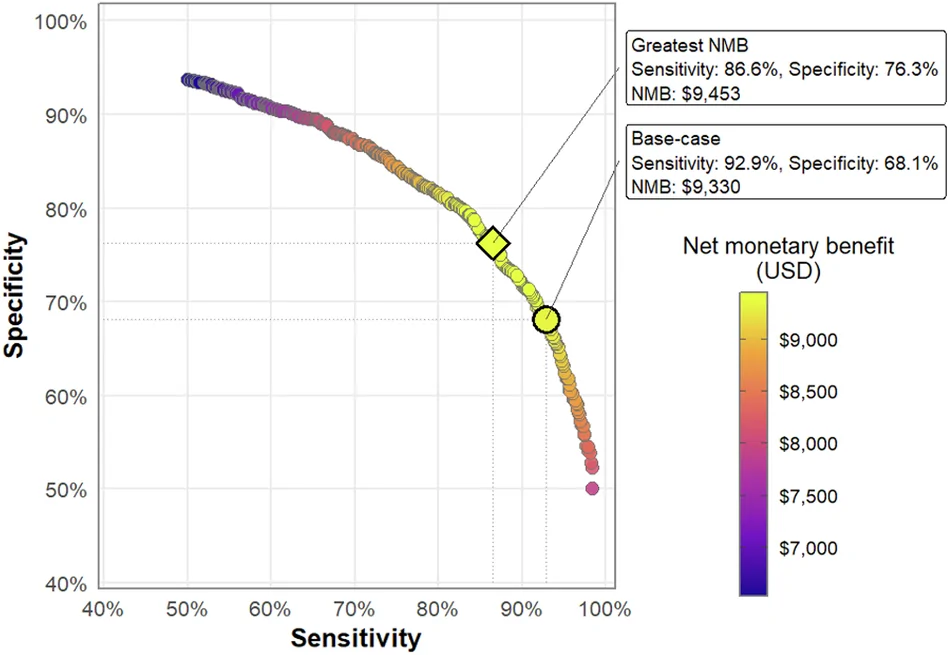
Net monetary benefit of different sensitivity and specificity combinations for opportunistic osteoporosis screening.Each point represents one of the 371 evaluated combinations of sensitivity and specificity. Point color indicates the corresponding net monetary benefit at a willingness-to-pay threshold of $39,492, representing one gross domestic product in Taiwan in 2025. Exact incremental cost-effectiveness ratio and net monetary benefits estimates for all diagnostic operating points are provided in Supplementary Table 1. All monetary values are presented in 2025 USD

The DL-based screening approach was considered cost-effective when the prevalence exceeded 14.3%. Regarding DL model performance, evaluation of 371 unique sensitivity and specificity combinations indicated that the greatest economic benefit was observed at a sensitivity of 86.6% and a specificity of 76.3%, yielding an NMB of 9,453 and an ICER of 20,319. As sensitivity increased toward 86.6% and specificity decreased toward 76.3% across different diagnostic combinations, the NMB generally increased and reached a peak, after which it slightly declined (Fig. 2 and Supplementary Table 1). At the base-case assumptions in a cohort of 1000 individuals, the operating point of 92.9% sensitivity and 68.1% specificity generated approximately 26 missed cases. In comparison, the economically optimal operating point generated approximately 48 missed cases. These findings indicate that threshold selection should consider not only cost-effective results, but also the acceptable number of missed cases in the intended clinical setting.

### Sensitivity analyses

**Fig. 3:**
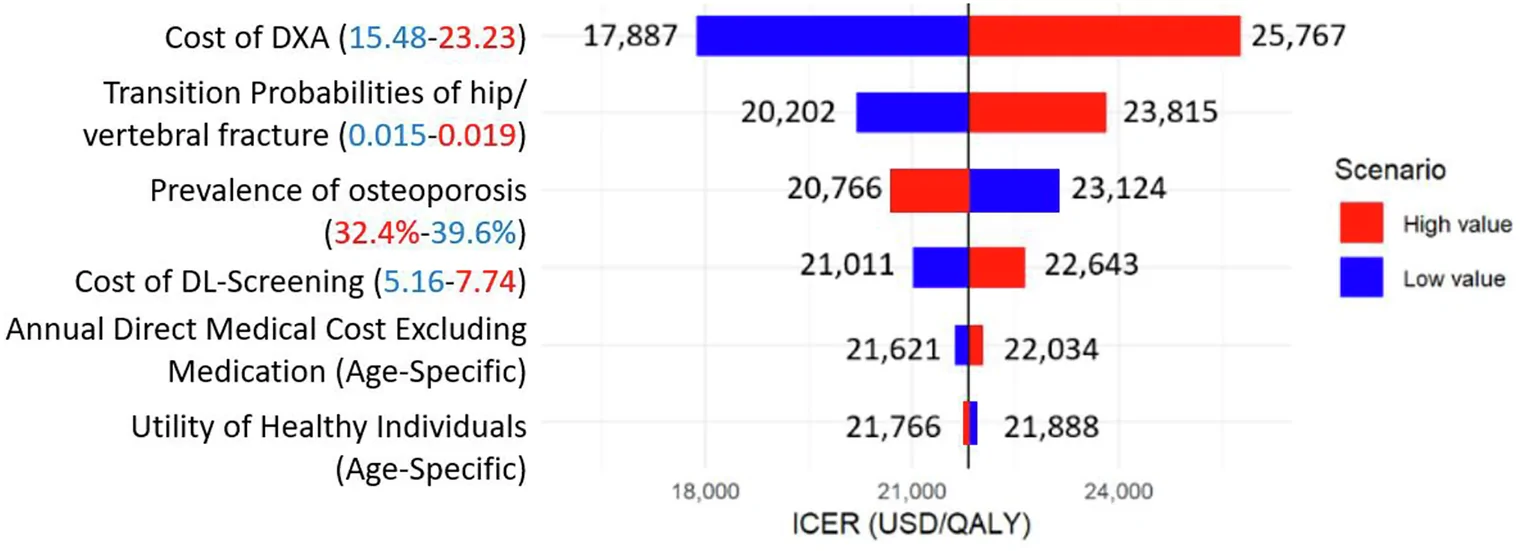
Tornado diagram of one-way sensitivity analysis. Red: Upper bound; Blue: Lower bound; WTP threshold was set at one GDP per capita. Abbreviations: DL, deep-learning; DXA, Dual-energy X-ray Absorptiometry; ICER, incremental cost-effectiveness ratio; WTP, willingness-to-pay; QALY, quality-adjusted life year

The one-way deterministic sensitivity analysis identified the cost of DXA as the most influential parameter affecting the ICER. The prevalence of osteoporosis and the incidence of hip or vertebral fracture, defined as the transition probabilities from a healthy state to hip fracture or vertebral fracture, were also influential. Additional influential parameters included the cost per DL-based screening approach, other direct medical costs excluding medication, and the utility of healthy individuals, although none of these parameters changed the primary conclusion (Fig. 3).

**Fig. 4:**
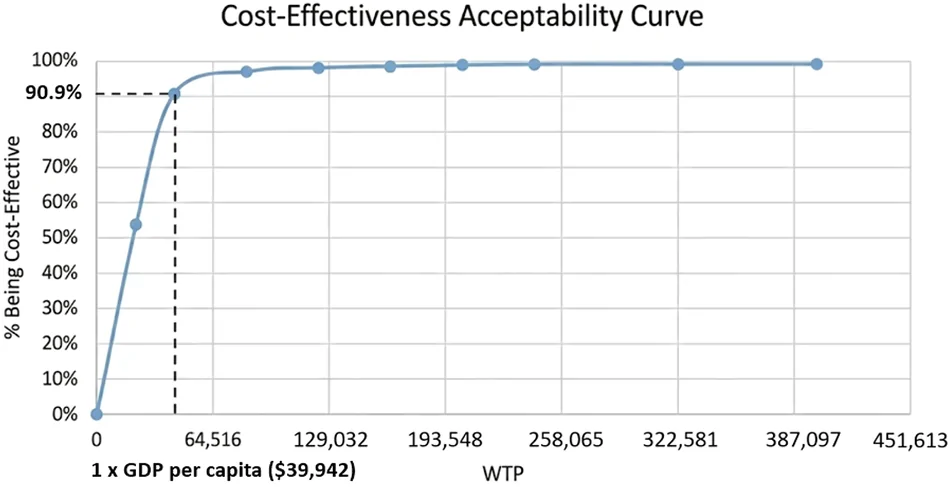
Cost-effectiveness acceptability curve of different WTP thresholds. Abbreviations: DL deep learning, WTP willingness-to-pay, QALY quality-adjusted life year

Probabilistic sensitivity analysis based on 5000 simulations, in which each model parameter was varied according to its respective distribution, indicated that the DL-based screening approach had a 90.9% probability of being cost-effective at a WTP threshold of one GDP per capita (Fig. 4 and Supplementary Fig. 1).

### Scenario analyses

Results of the scenario analysis showed that the economic value of the DL-based screening approach was influenced by both confirmatory DXA examination after positive DL screening and pharmacological treatment initiation after DXA-confirmed osteoporosis. In the one-way analysis of DXA examination, the most favorable outcome was observed under the base-case assumption that 100% of individuals with positive DL screening results received confirmatory DXA. As the proportion of DXA examinations decreased, the economic outcomes progressively worsened. DL-based screening remained cost-effective at a DXA examination rate of 20% (ICER 38,146), whereas the ICER exceeded the willingness-to-pay threshold at lower rates, including the OPSCAN-observed rate of 14.6% (ICER 45,691) **(**Table 2**)**. In the two-way scenario analysis, lower DXA examination and lower pharmacological treatment initiation further reduced the economic value of DL-based screening. When the OPSCAN-observed DXA examination rate of 14.6% and the Taiwan-based treatment initiation rate of 28.7% were applied, the approach was no longer cost-effective (Table 3) [13, 33].

**Table 2:** One-way scenario analysis of the confirmatory DXA examination rate

Confirmatory DXA examination rate	Cost (USD)	QALYs	ICER (USD/QALY)
10%	4324	26.511	58,546
14.6%ᵃ	4756	26.536	45,691
20%	5263	26.564	**38,146**
40%	7141	26.670	**27,947**
50%	8080	26.723	**25,907**
60%	9020	26.776	**24,547**
80%	10,898	26.882	**22,847**
100% (base-case)	12,776	26.990	**21,827**

All scenarios assumed complete pharmacological treatment initiation following DXA-confirmed osteoporosis. Costs and QALYs are reported per person for the DL-based screening approach; the no-screening comparator was associated with a cost of USD 1,226 and 26.460 QALYs. ICERs were calculated against no screening. The confirmatory DXA examination rate varied from 10% to 100%.

ICERs marked in bold represent cost-effective outcomes under the willingness-to-pay threshold of one GDP per capita.

*DL* deep learning, *DXA* dual-energy X-ray absorptiometry, *ICER* incremental cost-effectiveness ratio, *QALY* quality-adjusted life-year

ᵃ Confirmatory DXA examination rate observed in the OPSCAN trial [13]

**Table 3:** Two-way scenario analysis of confirmatory DXA examination rate and pharmacological treatment initiation rate

	Pharmacological treatment initiation rate				
Confirmatory DXA examination rate	20%	28.7%ᵃ	50%	75%	100%
10%	300,536	208,841	119,043	78,712	58,546
14.6%ᵇ	236,265	164,053	93,335	61,573	45,691
20%	198,540	137,764	78,245	51,513	**38,146**
40%	147,542	102,225	57,846	**37,913**	**27,947**
50%	137,342	95,117	53,766	**35,193**	**25,907**
60%	130,543	90,379	51,046	**33,380**	**24,547**
80%	122,043	84,456	47,646	**31,113**	**22,847**
100%	116,943	80,902	45,606	**29,753**	**21,827**

*DXA* dual-energy X-ray absorptiometry, *ICER* incremental cost-effectiveness ratio, *QALY* quality-adjusted life-year

Values are incremental cost-effectiveness ratios (ICERs) in USD per QALY, compared with no screening. Shaded cells indicate combinations for which the ICER fell below the willingness-to-pay threshold of USD 39,492, corresponding to one GDP per capita in Taiwan. The confirmatory DXA examination rate varied from 10% to 100%, and the pharmacological treatment initiation rate from 20% to 100%.

ICERs marked in bold represent cost-effective outcomes under the willingness-to-pay threshold of one GDP per capita.

ᵃ Taiwan-based estimate of pharmacological treatment initiation [33]

ᵇ Confirmatory DXA examination rate observed in the OPSCAN trial [13]

## Discussion

We evaluated the economic value of a DL-based screening approach that utilizes existing CXRs for opportunistic osteoporosis screening among Taiwanese postmenopausal women. The base-case analysis showed that this approach was cost-effective compared with no screening. The DL-based screening approach became cost-effective when the osteoporosis prevalence exceeded 14.3%. Additionally, a diagnostic accuracy analysis varying sensitivity and specificity identified parameter combinations that yielded a more favorable ICER of 20,319.

Economic evaluations of DL-based opportunistic osteoporosis screening using CXR conducted in multiple countries, including Japan, South Korea, Singapore, the United States, and Germany, have consistently reported that this approach is cost-effective under their respective modeling assumptions [14–18]. Some of these studies also reveal a clear association between disease prevalence and cost-effective outcomes. In South Korea, where fixed prevalence inputs of approximately 37% in women and 7.5% in men were used, DL-based screening demonstrated more favorable economic outcomes among women with higher prevalence than among men with lower prevalence [15]. In Japan, analyses conducted under multiple prevalence scenarios, all exceeding 10%, reported cost-effective or dominant results across these scenarios [14]. Similarly, in Singapore, where baseline prevalence was reported at about 17.3% for women aged 50 years and older, DL-based screening was found to be cost-effective across several prevalence levels examined [16]. The prevalence applied in our analysis (36%) falls within the range reported in these international studies and supports the observed economic value of the DL-based screening approach [34]. Our study extends this evidence by quantifying the prevalence level above which the approach becomes economically favorable in the Taiwanese setting.

The analysis of sensitivity and specificity combinations demonstrated an important trade-off between diagnostic detection and economic efficiency. Although greater sensitivity reduces the number of missed osteoporosis cases, lower specificity increases false positive results, DXA examinations, and healthcare expenditure. This issue may be particularly important in populations with lower osteoporosis prevalence, where false positive results constitute a larger proportion of positive screening results [35–37]. Evidence from an economic evaluation of ophthalmologic screening programs has similarly demonstrated that optimal diagnostic thresholds may differ across populations because of variations in disease prevalence and healthcare resource availability, emphasizing the need to balance sensitivity and specificity according to the local context [38]. The selection of diagnostic performance for DL-based osteoporosis screening should therefore consider not only the accuracy, but also the acceptable number of missed cases and the economic consequences of confirmatory testing.

The economic value of this approach ultimately depends on how positive screening results are translated into clinical action. In a feasible implementation pathway, the DL model would be applied to existing CXRs within the routine radiology or electronic medical record workflow, and positive results would be flagged for review by the responsible physician or an osteoporosis care coordinator. Patients identified as screen-positive would be informed that the result indicates elevated osteoporosis risk rather than a definitive diagnosis, and would be referred for confirmatory DXA. Where onsite DXA is available, the examination could be scheduled within the same institution; where it is not, referral could be arranged through regional hospitals, osteoporosis clinics, or contracted facilities. Pharmacological treatment would then be considered only after DXA-confirmed osteoporosis and subsequent clinical evaluation.

Real-world evidence from the OPSCAN trial indicates that DXA completion following DL-based high-risk notification may be limited. In the trial, 12.8% of high-risk participants underwent the fully reimbursed DXA examination offered by the study, while an additional 1.8% underwent DXA arranged by their primary care physicians, resulting in an overall completion rate of 14.6% [12, 13]. Consistent with these findings, our scenario analyses showed that lower DXA completion reduced the economic value of the screening approach. When Taiwan-based estimates for both DXA completion and pharmacological treatment initiation were applied simultaneously, the approach did not meet the prespecified WTP threshold. These findings indicate that economic value is not determined solely by diagnostic performance, but also by the healthcare system’s ability to complete the post-screening care cascade. Follow-up programs such as patient notification or regional coordination therefore represent key targets for improving real-world implementation.

A notable strength of this study is the systematic evaluation of a wide range of sensitivity and specificity combinations across the diagnostic continuum. To our knowledge, this is the first economic evaluation of DL-based opportunistic osteoporosis screening to examine how diagnostic threshold selection affects both economic value and missed case outcomes. In addition, the analysis incorporated extensive scenario and sensitivity analyses, including variations in osteoporosis prevalence, DXA examination, and treatment initiation rates, allowing the assessment of the economic value under different implementation conditions. These findings therefore contribute to the growing international evidence base on the economic implications of DL-based opportunistic osteoporosis screening and provide context for comparison with studies conducted in other healthcare systems.

Several limitations should be acknowledged. First, although model inputs were preferentially obtained from Taiwan-specific sources, some parameters were derived from multiple studies or comparable settings, which may introduce uncertainty despite the robustness observed in sensitivity analyses. Second, the base-case assumptions of complete confirmatory DXA examination and complete pharmacological treatment initiation may not reflect real-world implementation. Although alternative scenario analyses were conducted using lower examination and treatment initiation rates reported in Taiwan, the availability of implementation data remains limited, and actual practice patterns may vary across healthcare settings. Third, treatment effects were represented using medication class and persistence-specific hazard ratios. Changes in adherence, delayed dosing, treatment switching, or reinitiation are not simulated. Routine monitoring costs and adverse event costs were also not included. These simplifications may overestimate the effectiveness or underestimate the costs of pharmacological treatment under routine clinical practice. Fourth, osteoporosis prevalence was derived from claims data and may underestimate the true burden because of underdiagnosis, while annual fracture incidence was applied as a fixed transition probability. because suitable age-specific Taiwan data were unavailable. As fracture risk rises with age, this simplification may underestimate fracture events in later cycles, although its influence was examined in the deterministic sensitivity analysis. Finally, the cohort Markov structure did not capture patient-level heterogeneity, individual fracture history, or the cumulative effects of sequential fractures. These limitations should be considered when interpreting the long-term clinical and economic outcomes, and future studies incorporating prospective implementation data and real-world treatment pathways are warranted.

## Conclusion

This study evaluated the economic value of a DL-based opportunistic osteoporosis screening approach using existing CXRs in Taiwanese postmenopausal women. Under the base-case assumption of full completion of the downstream care pathway, the approach was cost-effective. The economic value was closely linked to epidemiological conditions and to the selected sensitivity and specificity thresholds, and it was not maintained when the confirmatory DXA and pharmacological treatment initiation rates observed in Taiwan were applied simultaneously. Careful consideration of diagnostic performance, disease prevalence, and the capacity of the health system to complete the post-screening care cascade is therefore essential when interpreting economic results and planning implementation strategies.

## Supplementary information

Supplementary Information

## Data Availability

The data that support the findings of this study are openly available as Supplementary Materials. All supplementary materials can be found at the following link: https://github.com/ndmc41030018-cyber/CXR_osteoporosis_supplement/tree/main.
