# Supplementary material for: Cost-utility analysis of deep learning-based opportunistic osteoporosis screening using chest radiographs: implications of diagnostic thresholds and confirmatory testing
Source: Arch Osteoporos. 2026 Oct 23;21(1):149. doi: 10.1007/s11657-026-01788-5 (PMC13694338; doi:10.1007/s11657-026-01788-5)
Supplement: Supplementary file 1 [file 11657_2026_1788_MOESM1_ESM.docx]

**Supplementary Table 1**. Sensitivity and specificity combinations of the DL-model

| Sensitivity | Specificity | ΔCost | ΔQALY | ICER | NMB | Result |
| --- | --- | --- | --- | --- | --- | --- |
| 0.985061 | 0.5 | 441808 | 0.561119 | 787369.1 | 245143.2496 | cost-effective |
| 0.984127 | 0.522093 | 433084 | 0.560587 | 772555.1 | 253215.384 | cost-effective |
| 0.983193 | 0.527442 | 430793 | 0.560055 | 769197.5 | 254855.6077 | cost-effective |
| 0.98226 | 0.538837 | 426179 | 0.559523 | 761682.4 | 258818.4658 | cost-effective |
| 0.980392 | 0.54093 | 424902 | 0.55846 | 760845.7 | 258793.6257 | cost-effective |
| 0.979458 | 0.544186 | 423414 | 0.557928 | 758905.2 | 259629.8606 | cost-effective |
| 0.977591 | 0.545581 | 422405 | 0.556864 | 758542.1 | 259337.0242 | cost-effective |
| 0.975724 | 0.556977 | 417554 | 0.5558 | 751266.4 | 262885.4679 | cost-effective |
| 0.97479 | 0.558372 | 416781 | 0.555268 | 750594.4 | 263007.046 | cost-effective |
| 0.973856 | 0.566977 | 413239 | 0.554736 | 744929 | 265897.919 | cost-effective |
| 0.971989 | 0.56814 | 412319 | 0.553673 | 744698.4 | 265515.7505 | cost-effective |
| 0.971055 | 0.569767 | 411457 | 0.553141 | 743856 | 265726.6607 | cost-effective |
| 0.970121 | 0.572093 | 410327 | 0.552609 | 742527 | 266205.5672 | cost-effective |
| 0.969188 | 0.58 | 407053 | 0.552077 | 737312 | 268828.4439 | cost-effective |
| 0.968254 | 0.582093 | 406012 | 0.551545 | 736136.1 | 269218.0183 | cost-effective |
| 0.96732 | 0.584419 | 404882 | 0.551013 | 734795.8 | 269696.9247 | cost-effective |
| 0.966387 | 0.59 | 402502 | 0.550482 | 731181 | 271426.4805 | cost-effective |
| 0.965453 | 0.591395 | 401729 | 0.54995 | 730483 | 271548.0586 | cost-effective |
| 0.964519 | 0.594419 | 400331 | 0.549418 | 728645.6 | 272294.9614 | cost-effective |
| 0.963585 | 0.595116 | 399826 | 0.548886 | 728432.1 | 272148.5432 | cost-effective |
| 0.961718 | 0.595581 | 399174 | 0.547822 | 728656.2 | 271498.3785 | cost-effective |
| 0.960784 | 0.601395 | 396704 | 0.54729 | 724851.1 | 273317.2664 | cost-effective |
| 0.958917 | 0.604186 | 395159 | 0.546227 | 723433.4 | 273560.4226 | cost-effective |
| 0.957983 | 0.604884 | 394654 | 0.545695 | 723213.6 | 273414.0044 | cost-effective |
| 0.957049 | 0.611628 | 391826 | 0.545163 | 718732.9 | 275590.2206 | cost-effective |
| 0.956116 | 0.617209 | 389446 | 0.544631 | 715063.6 | 277319.7764 | cost-effective |
| 0.955182 | 0.617442 | 389120 | 0.544099 | 715163.3 | 276994.694 | cost-effective |
| 0.954248 | 0.617907 | 388704 | 0.543567 | 715098.9 | 276758.9438 | cost-effective |
| 0.953315 | 0.620233 | 387574 | 0.543035 | 713718.3 | 277237.8503 | cost-effective |
| 0.952381 | 0.621395 | 386891 | 0.542504 | 713158.4 | 277270.0963 | cost-effective |
| 0.951447 | 0.623953 | 385672 | 0.541972 | 711608.3 | 277838.3348 | cost-effective |
| 0.950514 | 0.63186 | 382398 | 0.54144 | 706260.5 | 280461.2115 | cost-effective |
| 0.94958 | 0.633023 | 381714 | 0.540908 | 705691.5 | 280493.4576 | cost-effective |
| 0.948646 | 0.633953 | 381120 | 0.540376 | 705286.8 | 280436.3715 | cost-effective |
| 0.947712 | 0.636977 | 379722 | 0.539844 | 703391.9 | 281183.2742 | cost-effective |
| 0.946779 | 0.641628 | 377699 | 0.539312 | 700333.9 | 282555.5016 | cost-effective |
| 0.945845 | 0.644186 | 376479 | 0.53878 | 698762 | 283123.7402 | cost-effective |
| 0.943978 | 0.651395 | 373237 | 0.537717 | 694113.7 | 285064.2062 | cost-effective |
| 0.943044 | 0.651628 | 372911 | 0.537185 | 694194 | 284739.1238 | cost-effective |
| 0.94211 | 0.654419 | 371602 | 0.536653 | 692443.4 | 285396.6945 | cost-effective |
| 0.941176 | 0.655581 | 370918 | 0.536121 | 691855.6 | 285428.9405 | cost-effective |
| 0.940243 | 0.657442 | 369967 | 0.535589 | 690766.3 | 285729.1828 | cost-effective |
| 0.939309 | 0.660698 | 368480 | 0.535057 | 688673.2 | 286565.4176 | cost-effective |
| 0.938375 | 0.66186 | 367796 | 0.534526 | 688079.9 | 286597.6636 | cost-effective |
| 0.936508 | 0.662558 | 367055 | 0.533462 | 688062.1 | 286036.831 | cost-effective |
| 0.935574 | 0.665349 | 365746 | 0.53293 | 686293.1 | 286694.4017 | cost-effective |
| 0.934641 | 0.670465 | 363544 | 0.532398 | 682842.6 | 288245.2933 | cost-effective |
| 0.932773 | 0.672791 | 362177 | 0.531334 | 681637.4 | 288309.7853 | cost-effective |
| 0.931839 | 0.673256 | 361762 | 0.530802 | 681537.8 | 288074.035 | cost-effective |
| 0.930906 | 0.675814 | 360543 | 0.530271 | 679921.8 | 288642.2736 | cost-effective |
| 0.929972 | 0.677209 | 359770 | 0.529739 | 679145.8 | 288763.8517 | cost-effective |
| 0.929038 | 0.679535 | 358640 | 0.529207 | 677693 | 289242.7582 | cost-effective |
| 0.928105 | 0.685581 | 356080 | 0.528675 | 673533.7 | 291150.9781 | cost-effective |
| 0.927171 | 0.687209 | 355218 | 0.528143 | 672579.8 | 291361.8883 | cost-effective |
| 0.926237 | 0.688605 | 354446 | 0.527611 | 671793.2 | 291483.4664 | cost-effective |
| 0.925303 | 0.688837 | 354120 | 0.527079 | 671852.5 | 291158.3841 | cost-effective |
| 0.923436 | 0.69 | 353200 | 0.526016 | 671462 | 290776.2156 | cost-effective |
| 0.922502 | 0.690698 | 352695 | 0.525484 | 671181.1 | 290629.7974 | cost-effective |
| 0.921569 | 0.69093 | 352369 | 0.524952 | 671240 | 290304.7151 | cost-effective |
| 0.920635 | 0.693023 | 351328 | 0.52442 | 669936.3 | 290694.2895 | cost-effective |
| 0.918768 | 0.693953 | 350497 | 0.523356 | 669710.5 | 290222.7889 | cost-effective |
| 0.917834 | 0.698372 | 348563 | 0.522824 | 666692.6 | 291505.6842 | cost-effective |
| 0.9169 | 0.70093 | 347344 | 0.522293 | 665036.9 | 292073.9228 | cost-effective |
| 0.915966 | 0.701163 | 347018 | 0.521761 | 665089.9 | 291748.8404 | cost-effective |
| 0.915033 | 0.703023 | 346066 | 0.521229 | 663943.3 | 292049.0827 | cost-effective |
| 0.914099 | 0.704419 | 345294 | 0.520697 | 663137.5 | 292170.6608 | cost-effective |
| 0.913165 | 0.706279 | 344342 | 0.520165 | 661986.5 | 292470.9031 | cost-effective |
| 0.912232 | 0.707209 | 343748 | 0.519633 | 661520.9 | 292413.817 | cost-effective |
| 0.911298 | 0.708605 | 342976 | 0.519101 | 660710.1 | 292535.3951 | cost-effective |
| 0.910364 | 0.710233 | 342113 | 0.51857 | 659725.4 | 292746.3053 | cost-effective |
| 0.90943 | 0.710698 | 341698 | 0.518038 | 659600.9 | 292510.5551 | cost-effective |
| 0.908497 | 0.712558 | 340747 | 0.517506 | 658440.4 | 292810.7974 | cost-effective |
| 0.907563 | 0.713023 | 340331 | 0.516974 | 658314.3 | 292575.0471 | cost-effective |
| 0.906629 | 0.713721 | 339827 | 0.516442 | 658015 | 292428.6289 | cost-effective |
| 0.904762 | 0.713953 | 339264 | 0.515378 | 658281.1 | 291689.1321 | cost-effective |
| 0.903828 | 0.714186 | 338938 | 0.514846 | 658327.9 | 291364.0497 | cost-effective |
| 0.902894 | 0.714419 | 338612 | 0.514315 | 658374.7 | 291038.9674 | cost-effective |
| 0.901961 | 0.715581 | 337928 | 0.513783 | 657726.1 | 291071.2134 | cost-effective |
| 0.900093 | 0.718837 | 336204 | 0.512719 | 655728.1 | 291493.0338 | cost-effective |
| 0.89916 | 0.72093 | 335164 | 0.512187 | 654377.1 | 291882.6082 | cost-effective |
| 0.898226 | 0.722093 | 334480 | 0.511655 | 653721.7 | 291914.8542 | cost-effective |
| 0.897292 | 0.723488 | 333707 | 0.511123 | 652890.1 | 292036.4323 | cost-effective |
| 0.896359 | 0.724186 | 333203 | 0.510592 | 652581.7 | 291890.0141 | cost-effective |
| 0.894491 | 0.727209 | 331568 | 0.509528 | 650735.7 | 292222.5024 | cost-effective |
| 0.893557 | 0.727442 | 331242 | 0.508996 | 650775.1 | 291897.42 | cost-effective |
| 0.892624 | 0.728605 | 330559 | 0.508464 | 650111.8 | 291929.6661 | cost-effective |
| 0.89169 | 0.729302 | 330054 | 0.507932 | 649798.9 | 291783.2479 | cost-effective |
| 0.890756 | 0.731163 | 329102 | 0.5074 | 648605 | 292083.4902 | cost-effective |
| 0.888889 | 0.732326 | 328182 | 0.506337 | 648150.4 | 291701.3217 | cost-effective |
| 0.887955 | 0.732791 | 327767 | 0.505805 | 648010.7 | 291465.5715 | cost-effective |
| 0.886088 | 0.733023 | 327204 | 0.504741 | 648261.4 | 290726.0746 | cost-effective |
| 0.885154 | 0.734186 | 326521 | 0.504209 | 647589.9 | 290758.3206 | cost-effective |
| 0.88422 | 0.734651 | 326105 | 0.503677 | 647449 | 290522.5704 | cost-effective |
| 0.883287 | 0.734884 | 325779 | 0.503145 | 647485.4 | 290197.488 | cost-effective |
| 0.882353 | 0.735116 | 325453 | 0.502614 | 647521.8 | 289872.4057 | cost-effective |
| 0.881419 | 0.735581 | 325038 | 0.502082 | 647380.4 | 289636.6554 | cost-effective |
| 0.880486 | 0.737674 | 323997 | 0.50155 | 645991.9 | 290026.2298 | cost-effective |
| 0.879552 | 0.73907 | 323224 | 0.501018 | 645135.4 | 290147.8079 | cost-effective |
| 0.876751 | 0.740465 | 321978 | 0.499422 | 644701.3 | 289440.5571 | cost-effective |
| 0.875817 | 0.743721 | 320491 | 0.49889 | 642407.3 | 290276.7919 | cost-effective |
| 0.874883 | 0.75 | 317842 | 0.498359 | 637778.1 | 292274.344 | cost-effective |
| 0.87395 | 0.750465 | 317427 | 0.497827 | 637625 | 292038.5937 | cost-effective |
| 0.872082 | 0.752326 | 316239 | 0.496763 | 636598.7 | 291924.4215 | cost-effective |
| 0.871148 | 0.753953 | 315377 | 0.496231 | 635543.8 | 292135.3317 | cost-effective |
| 0.870215 | 0.756977 | 313979 | 0.495699 | 633405.4 | 292882.2345 | cost-effective |
| 0.869281 | 0.757674 | 313474 | 0.495167 | 633066.5 | 292735.8163 | cost-effective |
| 0.868347 | 0.758372 | 312969 | 0.494636 | 632726.8 | 292589.3981 | cost-effective |
| 0.867414 | 0.76 | 312107 | 0.494104 | 631663.2 | 292800.3083 | cost-effective |
| 0.86648 | 0.760233 | 311781 | 0.493572 | 631683.3 | 292475.226 | cost-effective |
| 0.865546 | 0.762791 | 310562 | 0.49304 | 629891.5 | 293043.4645 | cost-effective |
| 0.863679 | 0.764419 | 309463 | 0.491976 | 629020.1 | 292839.9603 | cost-effective |
| 0.862745 | 0.764651 | 309137 | 0.491444 | 629037.4 | 292514.8779 | cost-effective |
| 0.861811 | 0.764884 | 308811 | 0.490912 | 629054.7 | 292189.7956 | cost-effective |
| 0.860878 | 0.766047 | 308127 | 0.490381 | 628343.4 | 292222.0416 | cost-effective |
| 0.859944 | 0.768372 | 306997 | 0.489849 | 626718.7 | 292700.948 | cost-effective |
| 0.85901 | 0.768605 | 306671 | 0.489317 | 626733.6 | 292375.8657 | cost-effective |
| 0.858077 | 0.769535 | 306077 | 0.488785 | 626200.2 | 292318.7796 | cost-effective |
| 0.855275 | 0.769767 | 305278 | 0.487189 | 626610 | 291164.8683 | cost-effective |
| 0.854342 | 0.770233 | 304862 | 0.486658 | 626441.3 | 290929.1181 | cost-effective |
| 0.853408 | 0.772093 | 303911 | 0.486126 | 625169.6 | 291229.3604 | cost-effective |
| 0.852474 | 0.772558 | 303496 | 0.485594 | 624998.9 | 290993.6101 | cost-effective |
| 0.851541 | 0.773256 | 302991 | 0.485062 | 624643.7 | 290847.1919 | cost-effective |
| 0.850607 | 0.774651 | 302218 | 0.48453 | 623734.6 | 290968.77 | cost-effective |
| 0.849673 | 0.774884 | 301892 | 0.483998 | 623746.3 | 290643.6877 | cost-effective |
| 0.848739 | 0.778605 | 300226 | 0.483466 | 620986.5 | 291658.5867 | cost-effective |
| 0.846872 | 0.779302 | 299485 | 0.482403 | 620818.8 | 291097.7541 | cost-effective |
| 0.845938 | 0.781395 | 298444 | 0.481871 | 619344.3 | 291487.3284 | cost-effective |
| 0.845005 | 0.782093 | 297939 | 0.481339 | 618980.1 | 291340.9103 | cost-effective |
| 0.844071 | 0.783488 | 297166 | 0.480807 | 618057.7 | 291462.4884 | cost-effective |
| 0.843137 | 0.786977 | 295590 | 0.480275 | 615459.2 | 292388.0553 | cost-effective |
| 0.842204 | 0.787907 | 294996 | 0.479743 | 614903.3 | 292330.9692 | cost-effective |
| 0.84127 | 0.788372 | 294580 | 0.479211 | 614718.9 | 292095.219 | cost-effective |
| 0.839402 | 0.78907 | 293839 | 0.478148 | 614535.8 | 291534.3863 | cost-effective |
| 0.838469 | 0.792093 | 292441 | 0.477616 | 612293 | 292281.2891 | cost-effective |
| 0.837535 | 0.792326 | 292115 | 0.477084 | 612292.2 | 291956.2067 | cost-effective |
| 0.836601 | 0.792558 | 291789 | 0.476552 | 612291.4 | 291631.1244 | cost-effective |
| 0.834734 | 0.792791 | 291226 | 0.475488 | 612477.6 | 290891.6275 | cost-effective |
| 0.8338 | 0.793721 | 290632 | 0.474957 | 611912.7 | 290834.5415 | cost-effective |
| 0.832866 | 0.795814 | 289591 | 0.474425 | 610405.1 | 291224.1158 | cost-effective |
| 0.831933 | 0.796047 | 289265 | 0.473893 | 610402.1 | 290899.0335 | cost-effective |
| 0.830999 | 0.797442 | 288492 | 0.473361 | 609455.5 | 291020.6116 | cost-effective |
| 0.829132 | 0.797907 | 287840 | 0.472297 | 609447.5 | 290370.4469 | cost-effective |
| 0.828198 | 0.798605 | 287336 | 0.471765 | 609064.7 | 290224.0287 | cost-effective |
| 0.827264 | 0.799767 | 286652 | 0.471233 | 608301.9 | 290256.2747 | cost-effective |
| 0.826331 | 0.800698 | 286058 | 0.470702 | 607727.2 | 290199.1886 | cost-effective |
| 0.825397 | 0.80093 | 285732 | 0.47017 | 607721.2 | 289874.1063 | cost-effective |
| 0.823529 | 0.80186 | 284901 | 0.469106 | 607328.3 | 289402.6057 | cost-effective |
| 0.822596 | 0.802791 | 284307 | 0.468574 | 606749.9 | 289345.5196 | cost-effective |
| 0.820728 | 0.803256 | 283655 | 0.46751 | 606735.5 | 288695.3549 | cost-effective |
| 0.819795 | 0.803721 | 283240 | 0.466979 | 606537.1 | 288459.6046 | cost-effective |
| 0.816993 | 0.803953 | 282440 | 0.465383 | 606898.7 | 287305.6934 | cost-effective |
| 0.81606 | 0.804651 | 281936 | 0.464851 | 606507.3 | 287159.2752 | cost-effective |
| 0.814192 | 0.804884 | 281373 | 0.463787 | 606684.9 | 286419.7784 | cost-effective |
| 0.813259 | 0.806512 | 280511 | 0.463255 | 605520.6 | 286630.6886 | cost-effective |
| 0.812325 | 0.807907 | 279738 | 0.462724 | 604546.7 | 286752.2667 | cost-effective |
| 0.811391 | 0.809535 | 278876 | 0.462192 | 603377.2 | 286963.1769 | cost-effective |
| 0.809524 | 0.81 | 278224 | 0.461128 | 603354.9 | 286313.0122 | cost-effective |
| 0.80859 | 0.811163 | 277540 | 0.460596 | 602568 | 286345.2582 | cost-effective |
| 0.807656 | 0.811395 | 277214 | 0.460064 | 602555.9 | 286020.1758 | cost-effective |
| 0.805789 | 0.811628 | 276652 | 0.459001 | 602726.2 | 285280.679 | cost-effective |
| 0.803922 | 0.812791 | 275732 | 0.457937 | 602117 | 284898.5105 | cost-effective |
| 0.802054 | 0.813721 | 274901 | 0.456873 | 601700.5 | 284427.01 | cost-effective |
| 0.80112 | 0.815116 | 274128 | 0.456341 | 600708.5 | 284548.5881 | cost-effective |
| 0.800187 | 0.815349 | 273802 | 0.455809 | 600694.1 | 284223.5057 | cost-effective |
| 0.799253 | 0.815814 | 273387 | 0.455277 | 600483.5 | 283987.7555 | cost-effective |
| 0.797386 | 0.816047 | 272824 | 0.454214 | 600650.7 | 283248.2587 | cost-effective |
| 0.796452 | 0.816977 | 272230 | 0.453682 | 600045.5 | 283191.1726 | cost-effective |
| 0.795518 | 0.817442 | 271814 | 0.45315 | 599833.1 | 282955.4223 | cost-effective |
| 0.793651 | 0.818372 | 270984 | 0.452086 | 599406.8 | 282483.9218 | cost-effective |
| 0.791783 | 0.820233 | 269795 | 0.451023 | 598186.3 | 282369.7496 | cost-effective |
| 0.788982 | 0.820465 | 268996 | 0.449427 | 598531.1 | 281215.8383 | cost-effective |
| 0.787115 | 0.821395 | 268165 | 0.448363 | 598098.2 | 280744.3378 | cost-effective |
| 0.786181 | 0.821628 | 267839 | 0.447831 | 598080.4 | 280419.2554 | cost-effective |
| 0.785247 | 0.822093 | 267424 | 0.447299 | 597862.9 | 280183.5052 | cost-effective |
| 0.784314 | 0.822558 | 267008 | 0.446768 | 597644.9 | 279947.7549 | cost-effective |
| 0.78338 | 0.822791 | 266682 | 0.446236 | 597626.5 | 279622.6725 | cost-effective |
| 0.782446 | 0.823488 | 266178 | 0.445704 | 597207.3 | 279476.2544 | cost-effective |
| 0.780579 | 0.823721 | 265615 | 0.44464 | 597370.3 | 278736.7575 | cost-effective |
| 0.779645 | 0.823953 | 265289 | 0.444108 | 597351.5 | 278411.6752 | cost-effective |
| 0.778711 | 0.824884 | 264695 | 0.443576 | 596728.6 | 278354.5891 | cost-effective |
| 0.777778 | 0.827209 | 263565 | 0.443045 | 594894.3 | 278833.4956 | cost-effective |
| 0.77591 | 0.827907 | 262823 | 0.441981 | 594648.5 | 278272.6629 | cost-effective |
| 0.774977 | 0.82814 | 262497 | 0.441449 | 594626.3 | 277947.5806 | cost-effective |
| 0.774043 | 0.828605 | 262082 | 0.440917 | 594401.5 | 277711.8303 | cost-effective |
| 0.772176 | 0.828837 | 261519 | 0.439853 | 594559.5 | 276972.3335 | cost-effective |
| 0.771242 | 0.830465 | 260657 | 0.439321 | 593317.1 | 277183.2437 | cost-effective |
| 0.770308 | 0.83093 | 260242 | 0.43879 | 593089.6 | 276947.4934 | cost-effective |
| 0.768441 | 0.83186 | 259411 | 0.437726 | 592633 | 276475.9929 | cost-effective |
| 0.767507 | 0.832326 | 258995 | 0.437194 | 592403.8 | 276240.2426 | cost-effective |
| 0.766573 | 0.833488 | 258312 | 0.436662 | 591560.4 | 276272.4886 | cost-effective |
| 0.76564 | 0.833721 | 257986 | 0.43613 | 591534.2 | 275947.4063 | cost-effective |
| 0.764706 | 0.835116 | 257213 | 0.435598 | 590482.5 | 276068.9844 | cost-effective |
| 0.763772 | 0.835581 | 256798 | 0.435067 | 590249.6 | 275833.2341 | cost-effective |
| 0.762838 | 0.835814 | 256472 | 0.434535 | 590221.7 | 275508.1517 | cost-effective |
| 0.760037 | 0.836279 | 255583 | 0.432939 | 590343.9 | 274443.5726 | cost-effective |
| 0.759104 | 0.836512 | 255257 | 0.432407 | 590316 | 274118.4902 | cost-effective |
| 0.75817 | 0.836977 | 254842 | 0.431875 | 590081.2 | 273882.7399 | cost-effective |
| 0.757236 | 0.83814 | 254158 | 0.431343 | 589224.5 | 273914.9859 | cost-effective |
| 0.756303 | 0.83907 | 253564 | 0.430812 | 588573 | 273857.8999 | cost-effective |
| 0.755369 | 0.839767 | 253059 | 0.43028 | 588127.5 | 273711.4817 | cost-effective |
| 0.754435 | 0.840465 | 252555 | 0.429748 | 587680.9 | 273565.0635 | cost-effective |
| 0.753501 | 0.84093 | 252139 | 0.429216 | 587441.4 | 273329.3132 | cost-effective |
| 0.752568 | 0.843023 | 251099 | 0.428684 | 585742.5 | 273718.8876 | cost-effective |
| 0.751634 | 0.843488 | 250683 | 0.428152 | 585500 | 273483.1374 | cost-effective |
| 0.747899 | 0.844186 | 249468 | 0.426025 | 585572.1 | 272093.4758 | cost-effective |
| 0.746965 | 0.845349 | 248785 | 0.425493 | 584698 | 272125.7218 | cost-effective |
| 0.744164 | 0.846512 | 247628 | 0.423897 | 584169.8 | 271329.1389 | cost-effective |
| 0.743231 | 0.848605 | 246587 | 0.423365 | 582445.5 | 271718.7133 | cost-effective |
| 0.742297 | 0.848837 | 246261 | 0.422834 | 582407 | 271393.631 | cost-effective |
| 0.741363 | 0.850465 | 245399 | 0.422302 | 581099.2 | 271604.5411 | cost-effective |
| 0.739496 | 0.851163 | 244658 | 0.421238 | 580806.5 | 271043.7085 | cost-effective |
| 0.738562 | 0.852093 | 244064 | 0.420706 | 580128.7 | 270986.6224 | cost-effective |
| 0.737628 | 0.853023 | 243470 | 0.420174 | 579449.3 | 270929.5364 | cost-effective |
| 0.736695 | 0.854186 | 242786 | 0.419642 | 578555.2 | 270961.7824 | cost-effective |
| 0.735761 | 0.854651 | 242371 | 0.419111 | 578298.3 | 270726.0321 | cost-effective |
| 0.733894 | 0.854884 | 241808 | 0.418047 | 578423.5 | 269986.5353 | cost-effective |
| 0.732026 | 0.855349 | 241156 | 0.416983 | 578335.2 | 269336.3706 | cost-effective |
| 0.731092 | 0.855814 | 240741 | 0.416451 | 578076.4 | 269100.6203 | cost-effective |
| 0.729225 | 0.856744 | 239910 | 0.415387 | 577556.8 | 268629.1198 | cost-effective |
| 0.728291 | 0.857209 | 239494 | 0.414856 | 577295.9 | 268393.3695 | cost-effective |
| 0.727358 | 0.857907 | 238990 | 0.414324 | 576818.8 | 268246.9513 | cost-effective |
| 0.726424 | 0.85814 | 238664 | 0.413792 | 576772.3 | 267921.8689 | cost-effective |
| 0.72549 | 0.858372 | 238338 | 0.41326 | 576725.6 | 267596.7866 | cost-effective |
| 0.724556 | 0.858605 | 238012 | 0.412728 | 576678.8 | 267271.7042 | cost-effective |
| 0.723623 | 0.858837 | 237686 | 0.412196 | 576631.9 | 266946.6219 | cost-effective |
| 0.722689 | 0.86 | 237002 | 0.411664 | 575716.8 | 266978.8679 | cost-effective |
| 0.721755 | 0.861628 | 236140 | 0.411133 | 574364.8 | 267189.7781 | cost-effective |
| 0.719888 | 0.862558 | 235309 | 0.410069 | 573828.8 | 266718.2775 | cost-effective |
| 0.718954 | 0.863488 | 234715 | 0.409537 | 573123.5 | 266661.1914 | cost-effective |
| 0.718021 | 0.863721 | 234389 | 0.409005 | 573071.6 | 266336.1091 | cost-effective |
| 0.716153 | 0.865581 | 233201 | 0.407941 | 571653.5 | 266221.9369 | cost-effective |
| 0.714286 | 0.866512 | 232370 | 0.406878 | 571106.2 | 265750.4364 | cost-effective |
| 0.709617 | 0.866744 | 231097 | 0.404218 | 571714.3 | 263767.6962 | cost-effective |
| 0.70775 | 0.866977 | 230535 | 0.403155 | 571826.8 | 263028.1994 | cost-effective |
| 0.706816 | 0.867209 | 230209 | 0.402623 | 571772.4 | 262703.117 | cost-effective |
| 0.705882 | 0.867442 | 229882 | 0.402091 | 571717.8 | 262378.0347 | cost-effective |
| 0.704949 | 0.86814 | 229378 | 0.401559 | 571218.1 | 262231.6165 | cost-effective |
| 0.702148 | 0.868837 | 228400 | 0.399963 | 571051.3 | 261256.3694 | cost-effective |
| 0.701214 | 0.869302 | 227984 | 0.399432 | 570771.7 | 261020.6191 | cost-effective |
| 0.70028 | 0.870465 | 227301 | 0.3989 | 569819.6 | 261052.8651 | cost-effective |
| 0.699346 | 0.87093 | 226885 | 0.398368 | 569537.6 | 260817.1149 | cost-effective |
| 0.698413 | 0.872093 | 226202 | 0.397836 | 568581.3 | 260849.3609 | cost-effective |
| 0.697479 | 0.873488 | 225429 | 0.397304 | 567397.5 | 260970.939 | cost-effective |
| 0.693744 | 0.873953 | 224304 | 0.395177 | 567603.9 | 259491.9454 | cost-effective |
| 0.69281 | 0.874651 | 223799 | 0.394645 | 567090 | 259345.5272 | cost-effective |
| 0.691877 | 0.875116 | 223384 | 0.394113 | 566801.3 | 259109.7769 | cost-effective |
| 0.690943 | 0.875581 | 222968 | 0.393581 | 566511.8 | 258874.0266 | cost-effective |
| 0.690009 | 0.876279 | 222464 | 0.393049 | 565994.3 | 258727.6085 | cost-effective |
| 0.689076 | 0.876744 | 222048 | 0.392517 | 565703 | 258491.8582 | cost-effective |
| 0.686275 | 0.877442 | 221070 | 0.390922 | 565509.7 | 257516.6111 | cost-effective |
| 0.685341 | 0.877907 | 220655 | 0.39039 | 565216.2 | 257280.8608 | cost-effective |
| 0.684407 | 0.87814 | 220329 | 0.389858 | 565150.9 | 256955.7785 | cost-effective |
| 0.683473 | 0.878372 | 220003 | 0.389326 | 565085.5 | 256630.6961 | cost-effective |
| 0.681606 | 0.878605 | 219440 | 0.388262 | 565184.2 | 255891.1993 | cost-effective |
| 0.678805 | 0.87907 | 218551 | 0.386667 | 565217.7 | 254826.6201 | cost-effective |
| 0.677871 | 0.879302 | 218225 | 0.386135 | 565151.9 | 254501.5378 | cost-effective |
| 0.676937 | 0.879535 | 217899 | 0.385603 | 565085.8 | 254176.4554 | cost-effective |
| 0.676004 | 0.88 | 217483 | 0.385071 | 564787.6 | 253940.7051 | cost-effective |
| 0.67507 | 0.880465 | 217068 | 0.384539 | 564488.5 | 253704.9549 | cost-effective |
| 0.673203 | 0.881163 | 216327 | 0.383476 | 564120.9 | 253144.1222 | cost-effective |
| 0.672269 | 0.88186 | 215822 | 0.382944 | 563586.4 | 252997.7041 | cost-effective |
| 0.671335 | 0.883256 | 215049 | 0.382412 | 562349.6 | 253119.2822 | cost-effective |
| 0.670401 | 0.883488 | 214723 | 0.38188 | 562279 | 252794.1998 | cost-effective |
| 0.669468 | 0.884186 | 214218 | 0.381348 | 561739.7 | 252647.7816 | cost-effective |
| 0.668534 | 0.885116 | 213624 | 0.380816 | 560964.3 | 252590.6955 | cost-effective |
| 0.6676 | 0.887674 | 212405 | 0.380284 | 558542.4 | 253158.9341 | cost-effective |
| 0.666667 | 0.888605 | 211811 | 0.379752 | 557760.3 | 253101.848 | cost-effective |
| 0.664799 | 0.888837 | 211248 | 0.378689 | 557841 | 252362.3512 | cost-effective |
| 0.663866 | 0.88907 | 210922 | 0.378157 | 557763.3 | 252037.2689 | cost-effective |
| 0.661064 | 0.889302 | 210123 | 0.376561 | 558003.6 | 250883.3576 | cost-effective |
| 0.660131 | 0.89 | 209618 | 0.376029 | 557450.6 | 250736.9394 | cost-effective |
| 0.659197 | 0.89093 | 209024 | 0.375498 | 556658.1 | 250679.8533 | cost-effective |
| 0.658263 | 0.892326 | 208251 | 0.374966 | 555386.9 | 250801.4314 | cost-effective |
| 0.656396 | 0.892791 | 207599 | 0.373902 | 555222.9 | 250151.2667 | cost-effective |
| 0.655462 | 0.893256 | 207184 | 0.37337 | 554901.3 | 249915.5164 | cost-effective |
| 0.654528 | 0.893488 | 206857 | 0.372838 | 554818.4 | 249590.4341 | cost-effective |
| 0.653595 | 0.894186 | 206353 | 0.372306 | 554255.3 | 249444.0159 | cost-effective |
| 0.651727 | 0.894651 | 205701 | 0.371243 | 554086.9 | 248793.8512 | cost-effective |
| 0.64986 | 0.894884 | 205138 | 0.370179 | 554158.8 | 248054.3544 | cost-effective |
| 0.646125 | 0.895116 | 204102 | 0.368051 | 554546.6 | 246486.0286 | cost-effective |
| 0.644258 | 0.895349 | 203539 | 0.366988 | 554620.4 | 245746.5318 | cost-effective |
| 0.64239 | 0.895814 | 202887 | 0.365924 | 554450.6 | 245096.3671 | cost-effective |
| 0.640523 | 0.896047 | 202324 | 0.36486 | 554524.6 | 244356.8703 | cost-effective |
| 0.638655 | 0.896512 | 201672 | 0.363796 | 554353.5 | 243706.7056 | cost-effective |
| 0.634921 | 0.896744 | 200636 | 0.361669 | 554749.3 | 242138.3798 | cost-effective |
| 0.63212 | 0.89814 | 199389 | 0.360073 | 553746.7 | 241431.129 | cost-effective |
| 0.631186 | 0.898372 | 199063 | 0.359542 | 553659 | 241106.0467 | cost-effective |
| 0.630252 | 0.89907 | 198559 | 0.35901 | 553073.4 | 240959.6285 | cost-effective |
| 0.628385 | 0.899767 | 197817 | 0.357946 | 552645.6 | 240398.7959 | cost-effective |
| 0.627451 | 0.9 | 197491 | 0.357414 | 552555.7 | 240073.7135 | cost-effective |
| 0.625584 | 0.901163 | 196571 | 0.35635 | 551623.1 | 239691.5451 | cost-effective |
| 0.623716 | 0.901395 | 196008 | 0.355287 | 551690.6 | 238952.0482 | cost-effective |
| 0.622782 | 0.90186 | 195593 | 0.354755 | 551346.9 | 238716.298 | cost-effective |
| 0.621849 | 0.902558 | 195088 | 0.354223 | 550749.8 | 238569.8798 | cost-effective |
| 0.619048 | 0.902791 | 194289 | 0.352627 | 550974.6 | 237415.9685 | cost-effective |
| 0.615313 | 0.903023 | 193252 | 0.3505 | 551362.5 | 235847.6428 | cost-effective |
| 0.612512 | 0.903256 | 192453 | 0.348904 | 551592.5 | 234693.7315 | cost-effective |
| 0.611578 | 0.903488 | 192127 | 0.348372 | 551498.7 | 234368.6492 | cost-effective |
| 0.608777 | 0.903953 | 191238 | 0.346777 | 551473.1 | 233304.07 | cost-effective |
| 0.607843 | 0.904186 | 190912 | 0.346245 | 551378.6 | 232978.9876 | cost-effective |
| 0.605976 | 0.904884 | 190171 | 0.345181 | 550929.7 | 232418.155 | cost-effective |
| 0.604108 | 0.905581 | 189429 | 0.344117 | 550478.2 | 231857.3224 | cost-effective |
| 0.601307 | 0.905814 | 188630 | 0.342522 | 550708.3 | 230703.4111 | cost-effective |
| 0.59944 | 0.906512 | 187888 | 0.341458 | 550252.5 | 230142.5785 | cost-effective |
| 0.598506 | 0.907209 | 187383 | 0.340926 | 549630.5 | 229996.1603 | cost-effective |
| 0.597572 | 0.907907 | 186879 | 0.340394 | 549006.6 | 229849.7421 | cost-effective |
| 0.596639 | 0.908605 | 186374 | 0.339862 | 548380.7 | 229703.3239 | cost-effective |
| 0.594771 | 0.908837 | 185811 | 0.338799 | 548441.3 | 228963.8271 | cost-effective |
| 0.593838 | 0.90907 | 185485 | 0.338267 | 548339.7 | 228638.7448 | cost-effective |
| 0.592904 | 0.909535 | 185070 | 0.337735 | 547973.3 | 228402.9945 | cost-effective |
| 0.59197 | 0.909767 | 184744 | 0.337203 | 547870.7 | 228077.9121 | cost-effective |
| 0.591036 | 0.910233 | 184328 | 0.336671 | 547502.4 | 227842.1619 | cost-effective |
| 0.588235 | 0.910698 | 183440 | 0.335076 | 547456.9 | 226777.5827 | cost-effective |
| 0.587302 | 0.91093 | 183113 | 0.334544 | 547352.7 | 226452.5003 | cost-effective |
| 0.586368 | 0.911163 | 182787 | 0.334012 | 547248.1 | 226127.418 | cost-effective |
| 0.583567 | 0.911395 | 181988 | 0.332416 | 547469.7 | 224973.5067 | cost-effective |
| 0.581699 | 0.911628 | 181425 | 0.331353 | 547528.8 | 224234.0099 | cost-effective |
| 0.580766 | 0.91186 | 181099 | 0.330821 | 547423.5 | 223908.9275 | cost-effective |
| 0.577965 | 0.913256 | 179853 | 0.329225 | 546291.5 | 223201.6767 | cost-effective |
| 0.576097 | 0.913953 | 179111 | 0.328161 | 545802.9 | 222640.8441 | cost-effective |
| 0.57423 | 0.914884 | 178281 | 0.327098 | 545038 | 222169.3435 | cost-effective |
| 0.573296 | 0.915116 | 177955 | 0.326566 | 544927.2 | 221844.2612 | cost-effective |
| 0.571429 | 0.915349 | 177392 | 0.325502 | 544979.1 | 221104.7644 | cost-effective |
| 0.569561 | 0.915814 | 176740 | 0.324438 | 544755.9 | 220454.5996 | cost-effective |
| 0.568627 | 0.916047 | 176414 | 0.323907 | 544643.8 | 220129.5173 | cost-effective |
| 0.56676 | 0.916279 | 175851 | 0.322843 | 544695.2 | 219390.0205 | cost-effective |
| 0.565826 | 0.916512 | 175525 | 0.322311 | 544582.4 | 219064.9381 | cost-effective |
| 0.564893 | 0.916744 | 175199 | 0.321779 | 544469.2 | 218739.8557 | cost-effective |
| 0.563959 | 0.916977 | 174873 | 0.321247 | 544355.7 | 218414.7734 | cost-effective |
| 0.563025 | 0.917907 | 174279 | 0.320715 | 543406.2 | 218357.6873 | cost-effective |
| 0.561158 | 0.921395 | 172465 | 0.319652 | 539541.4 | 218868.8398 | cost-effective |
| 0.560224 | 0.922093 | 171961 | 0.31912 | 538859 | 218722.4216 | cost-effective |
| 0.558357 | 0.922326 | 171398 | 0.318056 | 538891.8 | 217982.9248 | cost-effective |
| 0.557423 | 0.922558 | 171072 | 0.317524 | 538767.6 | 217657.8424 | cost-effective |
| 0.555556 | 0.923023 | 170420 | 0.31646 | 538517.9 | 217007.6777 | cost-effective |
| 0.554622 | 0.923256 | 170094 | 0.315929 | 538392.4 | 216682.5953 | cost-effective |
| 0.551821 | 0.923488 | 169294 | 0.314333 | 538581.9 | 215528.6841 | cost-effective |
| 0.550887 | 0.924186 | 168789 | 0.313801 | 537886.3 | 215382.2659 | cost-effective |
| 0.549953 | 0.924419 | 168463 | 0.313269 | 537758.7 | 215057.1835 | cost-effective |
| 0.548086 | 0.924651 | 167900 | 0.312205 | 537788.4 | 214317.6867 | cost-effective |
| 0.546218 | 0.924884 | 167338 | 0.311142 | 537818.2 | 213578.1899 | cost-effective |
| 0.543417 | 0.925116 | 166538 | 0.309546 | 538007.6 | 212424.2786 | cost-effective |
| 0.542484 | 0.926512 | 165765 | 0.309014 | 536433.1 | 212545.8567 | cost-effective |
| 0.54155 | 0.926744 | 165439 | 0.308482 | 536301 | 212220.7744 | cost-effective |
| 0.540616 | 0.926977 | 165113 | 0.307951 | 536168.4 | 211895.692 | cost-effective |
| 0.538749 | 0.927442 | 164461 | 0.306887 | 535902 | 211245.5273 | cost-effective |
| 0.537815 | 0.927674 | 164135 | 0.306355 | 535768.1 | 210920.4449 | cost-effective |
| 0.536881 | 0.927907 | 163809 | 0.305823 | 535633.7 | 210595.3626 | cost-effective |
| 0.53408 | 0.92814 | 163010 | 0.304227 | 535815 | 209441.4513 | cost-effective |
| 0.533147 | 0.928372 | 162684 | 0.303696 | 535679.7 | 209116.3689 | cost-effective |
| 0.532213 | 0.929302 | 162090 | 0.303164 | 534660 | 209059.2829 | cost-effective |
| 0.530345 | 0.929535 | 161527 | 0.3021 | 534679.7 | 208319.786 | cost-effective |
| 0.529412 | 0.93 | 161111 | 0.301568 | 534245.3 | 208084.0358 | cost-effective |
| 0.528478 | 0.931395 | 160339 | 0.301036 | 532622.3 | 208205.6139 | cost-effective |
| 0.527544 | 0.931628 | 160013 | 0.300504 | 532480 | 207880.5315 | cost-effective |
| 0.526611 | 0.93186 | 159687 | 0.299973 | 532337.2 | 207555.4491 | cost-effective |
| 0.524743 | 0.932093 | 159124 | 0.298909 | 532348.8 | 206815.9523 | cost-effective |
| 0.52381 | 0.932326 | 158798 | 0.298377 | 532205 | 206490.87 | cost-effective |
| 0.522876 | 0.932558 | 158472 | 0.297845 | 532060.6 | 206165.7876 | cost-effective |
| 0.521942 | 0.932791 | 158146 | 0.297313 | 531915.8 | 205840.7052 | cost-effective |
| 0.520075 | 0.933721 | 157315 | 0.296249 | 531021.4 | 205369.2047 | cost-effective |
| 0.514472 | 0.933953 | 155805 | 0.293058 | 531652.4 | 202972.0501 | cost-effective |
| 0.513539 | 0.934186 | 155479 | 0.292526 | 531504.4 | 202646.9677 | cost-effective |
| 0.512605 | 0.934419 | 155153 | 0.291995 | 531355.9 | 202321.8854 | cost-effective |
| 0.511671 | 0.934651 | 154827 | 0.291463 | 531206.8 | 201996.803 | cost-effective |
| 0.510738 | 0.934884 | 154501 | 0.290931 | 531057.2 | 201671.7206 | cost-effective |
| 0.507937 | 0.935116 | 153701 | 0.289335 | 531222.6 | 200517.8094 | cost-effective |
| 0.507003 | 0.935581 | 153286 | 0.288803 | 530762.6 | 200282.0591 | cost-effective |
| 0.505135 | 0.936047 | 152634 | 0.28774 | 530458.4 | 199631.8944 | cost-effective |
| 0.503268 | 0.936279 | 152071 | 0.286676 | 530463.6 | 198892.3975 | cost-effective |
| 0.500467 | 0.937209 | 151004 | 0.28508 | 529688.1 | 198006.4826 | cost-effective |

Note: Costs and resulting ICER are presented in 2025 TWD.

**Supplementary Figure 1**. Decision tree of evaluating the cost-effectiveness of opportunistic osteoporosis screening using deep learning-based (DL-based) chest radiographs in Taiwanese postmenopausal women.

**Supplementary Figure 2**. Cost-effectiveness plane of base-case assumptions under 5,000 probabilistic simulations

Note: Green: cost-effective results; Red: non-cost-effective results; WTP threshold was set at $39,942, equaling to one GDP per capita in Taiwan; Costs were shown in 2025 USD.

Abbreviations: WTP, willingness-to-pay; QALY, quality-adjusted life year; TWD, New Taiwan Dollars
